# Aspirin Desensitization: Implications for Acetylsalicylic Acid-Sensitive Pregnant Women

**DOI:** 10.3390/medicina57040390

**Published:** 2021-04-17

**Authors:** Filipe Benito-Garcia, Inês Pires, Jorge Lima

**Affiliations:** 1 Immunoallergy Department, CUF Descobertas Hospital, 1998-018 Lisbon, Portugal; filipe.benito.garcia@gmail.com; 2 São Bernardo Hospital, Centro Hospitalar de Setúbal, 2910-549 Setúbal, Portugal; inesjgpires@gmail.com; 3 Department of Obstetrics and Gynecology, CUF Descobertas Hospital, 1998-018 Lisbon, Portugal; 4 Comprehensive Health Research Centre (CHRC), CEDOC, NOVA Medical School, Universidade Nova de Lisboa, 1169-056 Lisbon, Portugal

**Keywords:** antiphospholipid syndrome, Aspirin, Aspirin desensitization, fetal growth restriction, preeclampsia, pregnancy

## Abstract

Low-dose acetylsalicylic acid (ASA) is widely used during pregnancy to prevent obstetric complications of placental dysfunction, such as preeclampsia, stillbirth and fetal growth restriction, and obstetric complications in pregnant women with antiphospholipid syndrome. ASA-sensitive pregnant women cannot benefit from the effects of ASA due to the possibility of severe or potentially life-threatening hypersensitivity reactions to ASA. ASA desensitization is a valuable and safe therapeutic option for these women, allowing them to start daily prophylaxis with ASA and prevent pregnancy complications. The authors discuss the recent advances in obstetric conditions preventable by ASA and the management of ASA hypersensitivity in pregnancy, including ASA desensitization. To encourage the implementation of ASA desensitization protocols in ASA-sensitive pregnant women, they also propose a practical approach for use in daily clinical practice.

## 1. Introduction

Acetylsalicylic acid (ASA), commonly known as aspirin, is widely used during pregnancy to prevent obstetric complications of placental dysfunction, such as preeclampsia, stillbirth and fetal growth restriction (FGR), and obstetric complications in pregnant women with antiphospholipid syndrome (APLS) [1]. ASA and nonsteroidal anti-inflammatory drugs (NSAIDs) are the second most common causes of drug-induced hypersensitivity reactions, with ASA hypersensitivity affecting approximately 2% of the general population [2,3,4]. A cross-sectional survey of a general adult population self-reported NSAID hypersensitivity of 2.5% in women in the childbearing age [3]. With the generalized and widespread over-the-counter use of this class of drugs, the incidence of hypersensitivity reactions to NSAIDs is growing, and more women who become pregnant are confronted with this problem [5].

ASA-sensitive pregnant women with the aforementioned obstetric complications cannot benefit from the effects of ASA because of the possibility of an allergic reaction, which could be severe and life-threatening in some cases [5]. There are published cases of ASA-sensitive pregnant women who were successfully managed with an ASA desensitization protocol, allowing these women to start prophylactic treatment with ASA without triggering an allergic reaction [6,7,8].

To increase awareness of this problem, the authors reviewed the recent advances in the field of obstetric conditions preventable by ASA and the management of ASA hypersensitivity in pregnancy, including ASA desensitization. To assist obstetricians and allergists in the implementation of ASA desensitization protocols in ASA-sensitive pregnant women, the authors propose a practical approach for use in daily clinical practice.

## 2. Obstetric Complications Preventable by ASA

FGR, preeclampsia, and stillbirth are obstetric complications secondary to placental dysfunction that probably emerge early during pregnancy [9]. FGR occurs when a fetus does not reach its full growth potential, being the condition objectively defined as an estimated fetal weight under the 10th percentile [1,10]. Preeclampsia affects several organ systems and occurs in 2–8% [11] of pregnancies, being defined as high blood pressure (systolic 140 mmHg or higher or diastolic 90 mmHg or higher) with concomitant proteinuria (>0.3 g/24 h) diagnosed after 20 weeks of gestation [12]. According to the most recent classifications, the diagnosis of preeclampsia can be made when hypertension is present with maternal organ injury or placental insufficiency, even in the absence of proteinuria [13].

Optimal fetal growth depends on efficient gaseous and nutrient placental exchange, which is elicited by a low-resistance and high-flow uteroplacental circulation [10]. This uteroplacental circulation results from disruption of the smooth muscle layer and vascular remodeling led by the trophoblastic invasion of the uterine spiral arteries initiated at the 8th–10th weeks of gestation. This process is critical for a successful pregnancy. When trophoblast invasion is affected, there is a deficient remodeling of the spiral arteries and high-resistance and low-flow circulation persist, causing a reduced maternal blood supply to the placenta. This state of placental hypoxia and oxidative stress progressively results in a more extensive dysfunction of the trophoblast and potentiates a sequence of events that culminate in the aforementioned placenta-mediated complications of pregnancy: imbalance between angiogenic and antiangiogenic factors in maternal blood circulation, release of proinflammatory cytokines that leads to systemic endothelial cell dysfunction, imbalance in prostaglandin synthesis with increasing platelet thromboxane A_2_ (TXA_2_), and decreasing prostacyclin (PGI_2_) and platelet aggregation [9,14].

In the late 70s, the association between the regular administration of ASA during pregnancy and a lower probability of having preeclampsia was demonstrated [15]. Since then, numerous randomized studies have studied the role of the prophylactic use of ASA in the prevention of preeclampsia and contradictory results were reported [16]. It is only recently that robust studies have proven the beneficial effects of ASA on preeclampsia, FGR and other gestational complications. A systematic review and meta-analysis by Roberge et al. [17] found that starting ASA at ≤16 weeks of gestation strongly reduced the risk of preeclampsia by more than 60% and that 100 mg of ASA was more effective than lower doses. However, in a meta-analysis from the PARIS (Perinatal Antiplatelet Review of International Studies) Collaboration, Meher et al. [18] found that ASA reduced by 10% the risk of preeclampsia regardless of whether ASA was started at ≤ or >16 weeks of gestation. Both authors also found that low-dose ASA started before the 16th week of gestation moderately reduced the risk for developing FGR and stillbirth.

The ASPRE trial, a recent multicenter, double-blind, placebo-controlled trial reported that in women with singleton pregnancies at high risk for preeclampsia, the administration of ASA (150 mg/day) between 11–14 weeks until 36 weeks of pregnancy was associated with a 62% decrease in the incidence of early-onset preeclampsia (before 34 weeks of gestation) and an 89% reduction in early preeclampsia [16]. The risk of preeclampsia is defined by the presence of one or more high-risk factors (including history of preeclampsia, multifetal gestation, renal disease, type 1 or type 2 diabetes, autoimmune disease, and chronic hypertension) or more than one moderate-risk factor (including being the first pregnancy, a maternal age of 35 years or older, a body mass index greater than 30, family history of preeclampsia, personal history factors, and sociodemographic characteristics) [1].

In conclusion, the American College of Obstetricians and Gynecologists and the Society for Maternal-Fetal Medicine recommended low-dose ASA (81 mg/day) prophylaxis for women at high risk of preeclampsia, to be initiated between 12–28 weeks of gestation (being the optimal time before 16 weeks) with a daily administration until delivery. Low-dose ASA is also recommended to prevent early pregnancy loss, stillbirth, FGR, and preterm birth if high-risk factors for preeclampsia are present, but if they are not present, ASA is not recommended due to insufficient evidence [1].

APLS is an acquired autoimmune disorder defined as a prothrombotic condition that predisposes patients who are persistently positive for antiphospholipid antibodies (APL) to venous, arterial, or microvascular thrombosis in association with obstetric complications [19]. Obstetric APLS is characterized by recurrent early miscarriages (commonly earlier than 10th week of gestation) and an increased risk of conditions associated with ischemic placental dysfunction, such as early-onset preeclampsia, FGR, and fetal death [19]. Although criteria for classification of APLS have been proposed, the definition of clinically significant APL positivity is not well established because not every positive APL finding has diagnostic importance. Population-based studies are lacking, and consequently, the true incidence of APLS remains unknown [20].

APL promotes the activation of several cells, namely, endothelial, monocytes, and platelets, leading to the excessive production of tissue factors and TXA_2_. All these factors associated with the characteristic changes in hemostasis during pregnancy, lead to a prothrombotic state [21]. Therefore, the association between APL and thrombus formation in the arterial and/or venous vasculature suggests that obstetric morbidity related to APLS is secondary to poor vascular perfusion, being a consequence of placental dysfunction and infarction due to inflammatory and thrombotic changes [22].

**Figure 1:**
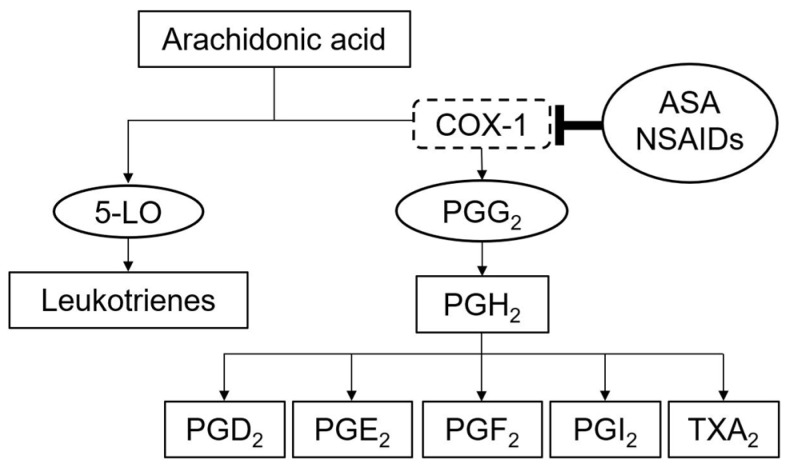
Mechanism of cyclooxygenase 1 (COX-1) inhibition by acetylsalicylic acid (ASA) or nonsteroidal anti-inflammatory drug (NSAID). 5-LO—5-lipoxygenase, PG—prostaglandin (types G_2_, H_2_, D_2_, E_2_, I_2_ and F_2_), TxA_2_—thromboxane A_2_.

Through the inhibition of thromboxane production (Figure 1), ASA reduces the risk of platelet-mediated vascular thrombosis [22]. Therefore, women with APLS are recommended to start low-dose ASA (75–100 mg daily) before pregnancy and to maintain it throughout pregnancy, which may increase the probability of pregnancy and embryo implantation, and also the achievement of successful live births in more than 70% of gestations [22,23]. In addition, anticoagulation therapy with low-molecular-weight heparin (LMWH) is also suggested by the recent European League Against Rheumatism recommendations [23]. Due to their embryopathic effects and fetal toxicity, oral anticoagulants must be suspended as soon as possible after pregnancy confirmation (within the first 6 weeks of gestation) and should be replaced by this double therapy with low-dose ASA and LMWH [24].

## 3. ASA Hypersensitivity

ASA and other NSAIDs are over-the-counter and are among the most consumed drugs worldwide, becoming one of the most frequent pharmacological groups involved in adverse reactions [5], which may vary from gastric discomfort, induced by well-known pharmacological side effects, to severe, possibly life-threatening hypersensitivity reactions [25].

Nonimmunological reactions that depend on the inhibition of the cyclooxygenase (COX)-1 pathway are the most frequent type. Immunological reactions often require drug-specific IgE production against the NSAID, which can induce both immunological and nonimmunological reactions in the same patient. COX-2 inhibitors are infrequently related to NSAID sensitivity due to the blockage of COX-1 isoenzyme only at high concentrations, not acting usually as a hapten for IgE-mediated reactions [26].

Some recent reports have considered NSAIDs as the major cause of drug-induced hypersensitivity, regardless of the severity of the reactions. The overall prevalence of this type of hypersensitivity ranges from 0.6–6%, depending on the type of reaction, population, and method of assessment [5]. NSAIDs are frequently involved in serious drug reactions, being responsible for more than half of all drug-induced anaphylaxis. Furthermore, up to 2% of deaths relate to an NSAID-induced fatal drug reaction/side effects, of which gastrointestinal hemorrhages are the main manifestations [27,28].

The diagnosis and management of NSAID hypersensitivity are not easy due to the association with undesirable dose-related effects, particularly in the gastrointestinal tract, which is crucial to distinguish from true hypersensitivity reactions [25].

NSAID hypersensitivity reactions are classified into five phenotypes characterized by their clinical manifestations, underlying allergic disease, cross-reactivity pattern with other COX-1 inhibitors, and distinct immunological or nonimmunological mechanisms [5]:

NSAID-exacerbated respiratory disease (NERD) is a distinct condition characterized by asthma, chronic rhinosinusitis with recurrent eosinophilic nasal polyps, and hypersensitivity reactions to all NSAIDs that inhibit COX-1. These drugs exacerbate clinical manifestations of the disease and are not the cause of the underlying respiratory inflammation [25]. The mechanisms of NERD were reviewed by Laidlaw and Boyce and involve abnormalities in the arachidonic acid pathway, promoting the systemic overproduction of proinflammatory eicosanoids, including cysteinyl leukotrienes and prostaglandin D_2_, and underproduction of or reduced response to the anti-inflammatory prostaglandin E_2_ (PGE_2_) [29].

NSAID-exacerbated cutaneous disease (NECD) is defined by urticaria and/or angioedema induced by NSAIDs in patients with underlying chronic spontaneous urticaria. Similar to NERD, the mechanism is believed to be correlated to the inhibition of COX-1 [30].

NSAID-induced urticaria/angioedema (NIUA) is characterized by the development of urticaria and/or angioedema after exposure to a COX-1 inhibitor in patients with no history of chronic urticaria and angioedema [25]. At least two NSAIDs belonging to different chemical groups are required to elicit symptoms [25].

Single-NSAID-induced urticaria/angioedema or anaphylaxis (SNIUAA) is an immediate hypersensitivity reaction, usually IgE-mediated, in which the patient reacts only to a single NSAID (or another drug in the same chemical group), presenting tolerance to others chemically nonrelated NSAIDs [5].

Single-NSAID-induced delayed reactions (SNIDR) are rare and include any reaction occurring after 24 h or more of exposure, involving any organ system ranging from mild cutaneous to systemic reactions. The underlying mechanism is thought to be T cell-mediated. Mild cutaneous reactions are maculopapular and fixed drug eruptions or contact dermatitis. Acute generalized exanthematous pustulosis, Stevens-Johnson syndrome/toxic epidermal necrolysis, and drug reaction with eosinophilia and systemic symptoms (DRESS) are considered severe reactions with the potential of being life-threatening [5].

NERD, NECD, and NIUA have a nonimmunological mechanism associated with COX-1 inhibition, and treatments including ASA desensitization and daily ASA therapy are recommended therapeutic options that provide positive long-term effects [31,32,33]. SNIUAA and SNIDR have an immunological mechanism, and there has been no documented successful ASA desensitization in these patients [25].

## 4. Diagnosis of ASA and NSAID Hypersensitivity Reactions

When there is a suspicion of NSAID hypersensitivity, the first thing to consider is a detailed clinical history. The management and diagnosis relies on the suspected or confirmed mechanism of the reaction. Skin testing, which includes skin prick and intradermal tests, should be considered only in suspected IgE-mediated reactions. It is only recommended for two NSAIDs (metamizole or paracetamol), as they are the only ones presenting good predictive values; however, these tests, if possible, should be postponed after labor [5,34].

The oral provocation test (OPT) is the gold standard for the confirmation or exclusion of the diagnosis, as well as to investigate which alternative NSAIDs can be safely used.

This procedure consists of the administration of increasing doses up to the therapeutic dose and observation for possible hypersensitive reactions. These reactions, especially the severe ones, occur during the first 4 hours after drug intake (immediate response). OPT is not recommended in cases of a severe anaphylactic reaction, severe medical or surgical condition, pregnancy, uncontrolled underlying chronic disease (asthma, urticaria), airway obstruction, or severe delayed type reactions (only patients with maculopapular and fixed drug eruptions can be tested). Due to its risk, OPT should always be performed at a center with experienced personnel, under cardiorespiratory surveillance, and with lung functional assessment. OPT is not recommended during pregnancy, and this procedure should be postponed until after labor to confirm or exclude the diagnosis [34].

## 5. ASA Desensitization Therapy and Pregnancy

ASA desensitization induces a temporary state of tolerance of ASA in ASA-sensitive patients. This is achieved by taking increasing doses of ASA over a short period of time (from several hours to a few days) until reaching the total cumulative therapeutic dose, which is tolerated and then daily maintained [35]. It is a suitable option only in patients in whom alternative drugs are less effective or unavailable, like ASA-sensitive pregnant women with FGR, preeclampsia, stillbirth or APLS. The first report on ASA desensitization was by Widal et al. [36] in 1922, but it was only in 1984 that Stevenson et al. [37] first described this procedure. Subsequently, there were multiple publications on various desensitization protocols and routes of administration in the context of NERD [38,39], NECD, and NIUA [31,33]. This procedure is also indicated for the treatment and prophylaxis of cardiovascular diseases in ASA-sensitive patients, due to its outstanding clinical efficacy, safety, and cost-effectiveness of ASA [40].

In cases of hypersensitivity reactions related to COX-1 inhibition, the desensitization results in downregulation of cysteinyl leukotriene receptors decreased extracellular histamine and tryptase levels after mast cell stimulation and decreased leukotriene production [41].

Regarding IgE-mediated reactions, the precise mechanism of desensitization is not known. Gollapudi et al. [42] have suggested that the mechanism is similar to that of penicillin desensitization with the need for prior exposure and the presence of antibodies. Therefore, like penicillin desensitization, recurrent NSAID exposure leads to saturation of anti-NSAID IgE antibody sites on mast cells and basophils, blocking their activation and depleting intracellular mediators (histamine) [43].

Several desensitization protocols have been published, with times to completion varying from hours to days to weeks [31,35,44]. ASA desensitization requires a multidisciplinary approach that always involves an allergist [42]. It is a procedure with potential risk that must be performed in a supervised hospital setting, with specialized staff with experience in managing anaphylactic reactions. It is contraindicated in patients with systemic vasculitis, severe SNIDR, peptic ulcer disease or hemorrhagic disease [35,38,42,43].

Penicillin desensitization in pregnant women has been performed for many years, with many reports proving its safety and efficacy [35,45]; however, only a few reports on ASA desensitization have been published. Two case reports of effective desensitization to ASA in women with thrombophilia and APLS were published [7,8], but only one describes desensitization during pregnancy. Alijotas-Reig et al. [6] published a case series of four women diagnosed with APLS, three of whom were pregnant. These women were successfully desensitized to ASA and then started a daily single dose of ASA associated with subcutaneous enoxaparin throughout pregnancy, until full-term delivery. They had favorable pregnancy outcomes, with no reported side effects or complications.

Because the published case reports of pregnant women subjected to ASA desensitization are few, the safety of this procedure can only be extrapolated from the numerous cases performed in nonpregnant patients, and some authors have recommended desensitization as an acceptable alternative approach in pregnant women [39,44]. The authors hope that this review will be useful to ensure ASA-sensitive pregnant women do not miss out on the clinical benefit of ASA use, when indicated, and larger cohorts will appear in the future to reinforce the safety of ASA desensitization.

## 6. Practical Approach for ASA-Sensitive Pregnant Women

Prepregnancy and antenatal care are crucial, and low-dose ASA is indicated for prevention of preeclampsia, FGR, stillbirth or obstetric complications related to APLS, during pregnancy [1,23]. However, ASA-sensitive pregnant women with the aforementioned obstetric complications cannot benefit from the effects of ASA due to the possibility of severe or potentially life-threatening hypersensitivity reactions. ASA desensitization is a valued and safe therapeutic alternative for these women, allowing them to start daily prophylaxis with ASA and prevent pregnancy complications [6].

**Figure 2:**
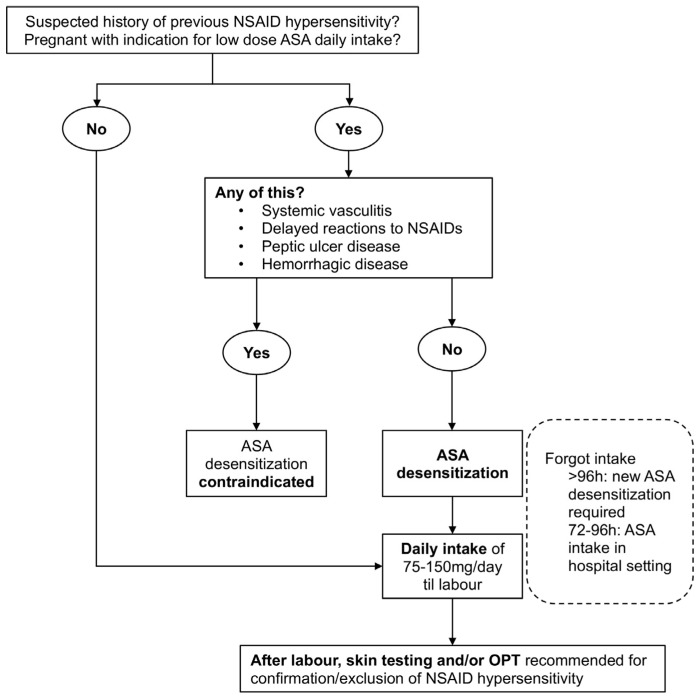
Therapeutic approach for ASA-sensitive pregnant women who have an indication for prophylactic treatment with low-dose ASA. ASA—acetylsalicylic acid; NSAIDs—nonsteroidal anti-inflammatory drugs; OPT—oral provocation test.

The authors propose an approach for ASA-sensitive pregnant women who need low-dose ASA (Figure 2). Pregnant women who need prophylactic treatment with low-dose ASA and are suspected of having NSAID hypersensitivity must be referred to an allergist for investigation. If indicated, ASA desensitization could be performed at any gestational age of pregnancy. From the obstetric view, this procedure should be initiated as soon as there is a clear clinical benefit for the obstetric complication in question.

A complete evaluation sustained on clinical manifestations, the timing of the reaction, reactions to other NSAIDs, and the presence of underlying disease allows the allergist to characterize pregnant women with a history of reaction to NSAIDs [5,34].

In nonpregnant patients, hypersensitivity to NSAIDs is usually clearly confirmed, as is the absence of an alternative drug. However, due to the risks of the hypersensitivity investigation in pregnant women and the urgent need to start low-dose ASA, desensitization can be started without clear confirmation of the hypersensitivity and only based on the suspicion of NSAID hypersensitivity. Skin tests and OPTs are not recommended for pregnant women, and prophylactic treatment with low-dose ASA and, consequently, ASA desensitization, should be started as soon as possible [35].

**Figure 3:**
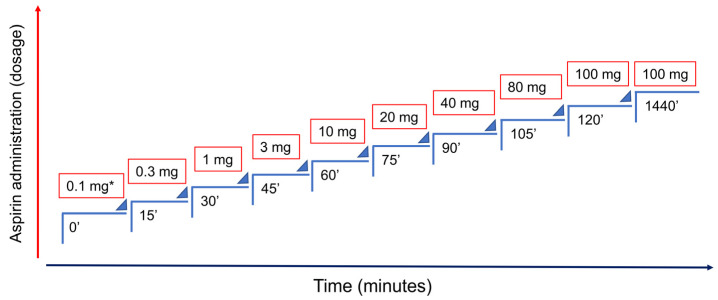
Aspirin desensitization protocol. The asterisk (*) represents the recommended starting dose for pregnant women who had a serious hypersensitivity reaction (anaphylaxis). If not, the protocol can be started at step 3 (1 mg). Adapted from Rossini et al. [40].

The authors have proposed an ASA desensitization protocol (Figure 3) based on previous protocols used [6,31] with increasing doses over 15-min intervals, beginning with a dose of 1 mg of ASA, until the cumulative target dose is achieved with tolerance. If the pregnant woman has a serious hypersensitivity reaction (anaphylaxis), the protocol should be started with 0.1 mg of ASA. The occurrence of drug-induced reactions is expected to be milder, shorter and self-limiting.

Once desensitized, the pregnant woman tolerate ASA for 2–3 days. If she forgets taking ASA for 1 day, it can be resumed at the previous dose. However, if more than 2 days have passed without taking ASA, the temporary state of tolerance is lost, and the risk of reaction with any COX-1 inhibition is higher. Therefore, if the pregnant woman has forgotten to take the ASA in the last 3/4 days, the ASA dose should be taken in a hospital facility under medical surveillance, and if more than 4 days have elapsed, new desensitization is required.

## 7. Conclusions

In ASA-sensitive pregnant women with obstetric complications preventable by daily intake of ASA, ASA desensitization could be a therapeutic option to allow these women to start ASA prophylaxis as soon as possible, with favorable pregnancy outcomes. Although there are few case reports in the literature concerning ASA desensitization in pregnant women, there are many reports of penicillin desensitization in pregnant women that report considerable safety.

NSAIDs are considered one of the most frequently reported drugs that induce hypersensitivity reactions, and the incidence of ASA-sensitive pregnant women is increasing. Although published data are scarce and more studies with more patients are necessary to ensure the safety of these patients under these challenging conditions, the benefits of treatment outrank the risks in ASA-sensitive pregnant women. To our knowledge, ASA desensitization is the best therapeutic option for ASA-sensitive pregnant women with obstetric complications preventable by daily ASA prophylaxis.
